# Supplementary material for: The impact of village heads’ educational levels on adolescent academic performance: Evidence from rural China
Source: PLoS One. 2025 Jan 2;20(1):e0315822. doi: 10.1371/journal.pone.0315822 (PMC11694985; doi:10.1371/journal.pone.0315822)
Supplement: Attachment — Submitted filename: PONE-D-24-18436-reviewer attachment.pdf [file pone.0315822.s001.pdf]

PLOS ONE

Educational Attainment of Village Heads and Adolescents' Academic Performance - A Perspective Based on the External Environment of Village Settlements

--Manuscript Draft--

Manuscript Number:	PONE-D-24-18436
Article Type:	Research Article
Full Title:	Educational Attainment of Village Heads and Adolescents' Academic Performance - A Perspective Based on the External Environment of Village Settlements
Short Title:	Educational Attainment of Village Heads and Adolescents' Academic Performance
Corresponding Author:	Jing Li<br>Hunan Institute of Engineering<br>xiangtan, hunan CHINA
Keywords:	Village chiefs' educational attainment; public goods provision; role modeling effect; adolescent education
Abstract:	This study investigates the relationship between the educational level of village heads and the academic performance of adolescents, utilizing data from the China Family Tracking Survey (CFPS). The analysis reveals that village chiefs with higher education levels significantly enhance the academic outcomes of adolescents within their communities. This positive effect persists even when controlling for endogeneity through the use of instrumental variables and conducting a variety of robustness checks. Further investigation indicates that these educated village leaders contribute to an increased provision of public goods, thereby improving the external environment of the village which supports academic performance. Additionally, well-educated village chiefs serve as role models within the village's social network, influencing parental educational aspirations and thereby augmenting adolescents' academic results. Notably, the impact of highly educated village chiefs is more pronounced for girls and adolescents from low-income families, highlighting its significance for promoting gender equity in education and breaking cycles of intergenerational poverty.
Order of Authors:	Jing Li
	Huan Deng
	Jun Li
Additional Information:	
Question	Response
**Financial Disclosure**<br><br>Enter a financial disclosure statement that describes the sources of funding for the work included in this submission. Review the submission guidelines for detailed requirements. View published research articles from *PLOS ONE* for specific examples.<br><br>This statement is required for submission and **will appear in the published article** if the submission is accepted. Please make sure it is accurate.	The author(s) received no specific funding for this work.

Powered by Editorial Manager® and ProduXion Manager® from Aries Systems Corporation

**Funded studies**<br>Enter a statement with the following details:<br>• Initials of the authors who received each award<br>• Grant numbers awarded to each author<br>• The full name of each funder<br>• URL of each funder website<br>• Did the sponsors or funders play any role in the study design, data collection and analysis, decision to publish, or preparation of the manuscript?<br><br>Did you receive funding for this work?	
**Competing Interests**<br><br>Use the instructions below to enter a competing interest statement for this submission. On behalf of all authors, disclose any competing interests that could be perceived to bias this work—acknowledging all financial support and any other relevant financial or non-financial competing interests.<br><br>This statement is required for submission and **will appear in the published article** if the submission is accepted. Please make sure it is accurate and that any funding sources listed in your Funding Information later in the submission form are also declared in your Financial Disclosure statement.<br><br>View published research articles from *PLOS ONE* for specific examples.	The authors have declared that no competing interests exist.

Powered by Editorial Manager® and ProduXion Manager® from Aries Systems Corporation

**NO authors have competing interests**<br><br>Enter: *The authors have declared that no competing interests exist.*<br><br>**Authors with competing interests**<br><br>Enter competing interest details beginning with this statement:<br><br>*I have read the journal's policy and the authors of this manuscript have the following competing interests: [insert competing interests here]*<br><br>* typeset	
**Ethics Statement**<br><br>Enter an ethics statement for this submission. This statement is required if the study involved:<br><br>• Human participants<br>• Human specimens or tissue<br>• Vertebrate animals or cephalopods<br>• Vertebrate embryos or tissues<br>• Field research<br><br>Write "N/A" if the submission does not require an ethics statement.<br><br>General guidance is provided below. Consult the submission guidelines for detailed instructions. **Make sure that all information entered here is included in the Methods section of the manuscript.**	N/A

Powered by Editorial Manager® and ProduXion Manager® from Aries Systems Corporation

**Format for specific study types**<br><br>**Human Subject Research (involving human participants and/or tissue)**<br>• Give the name of the institutional review board or ethics committee that approved the study<br>• Include the approval number and/or a statement indicating approval of this research<br>• Indicate the form of consent obtained (written/oral) or the reason that consent was not obtained (e.g. the data were analyzed anonymously)<br><br>**Animal Research (involving vertebrate animals, embryos or tissues)**<br>• Provide the name of the Institutional Animal Care and Use Committee (IACUC) or other relevant ethics board that reviewed the study protocol, and indicate whether they approved this research or granted a formal waiver of ethical approval<br>• Include an approval number if one was obtained<br>• If the study involved *non-human primates*, add *additional details* about animal welfare and steps taken to ameliorate suffering<br>• If anesthesia, euthanasia, or any kind of animal sacrifice is part of the study, include briefly which substances and/or methods were applied<br><br>**Field Research**<br><br>Include the following details if this study involves the collection of plant, animal, or other materials from a natural setting:<br>• Field permit number<br>• Name of the institution or relevant body that granted permission	
**Data Availability**<br><br>Authors are required to make all data underlying the findings described fully available, without restriction, and from the time of publication. PLOS allows rare exceptions to address legal and ethical concerns. See the PLOS Data Policy and FAQ for detailed information.	Yes - all data are fully available without restriction

A Data Availability Statement describing where the data can be found is required at submission. Your answers to this question constitute the Data Availability Statement and **will be published in the article**, if accepted.<br><br>**Important:** Stating 'data available on request from the author' is not sufficient. If your data are only available upon request, select 'No' for the first question and explain your exceptional situation in the text box.<br><br>Do the authors confirm that all data underlying the findings described in their manuscript are fully available without restriction?	
**Describe where the data may be found in full sentences. If you are copying our sample text, replace any instances of XXX with the appropriate details.**<br><br>• If the data are **held or will be held in a public repository**, include URLs, accession numbers or DOIs. If this information will only be available after acceptance, indicate this by ticking the box below. For example: *All XXX files are available from the XXX database (accession number(s) XXX, XXX.).*<br>• If the data are all contained **within the manuscript and/or Supporting Information files**, enter the following: *All relevant data are within the manuscript and its Supporting Information files.*<br>• If neither of these applies but you are able to provide **details of access elsewhere**, with or without limitations, please do so. For example:<br><br>*Data cannot be shared publicly because of [XXX]. Data are available from the XXX Institutional Data Access / Ethics Committee (contact via XXX) for researchers who meet the criteria for access to confidential data.*<br><br>*The data underlying the results presented in the study are available from (include the name of the third party*	The data is available for download from Peking University: https://opendata.pku.edu.cn/dataset.xhtml?persistentId=doi:10.18170/DVN/45LCSO

and contact information or URL).<br>• This text is appropriate if the data are owned by a third party and authors do not have permission to share the data.<br><br>* typeset	
Additional data availability information:	

Manuscript Click here to access/download;Manuscript;manuscript3.docx

Educational Attainment of Village Heads and Adolescents' Academic Performance - A Perspective Based on the External Environment of Village Settlements

Jing Li[1¶,#a,*]

(lijingcssci@foxmail.com)

Deng Huan[2¶,#a]

(454642713@qq.com)

Jun Li[3&,#b]

(lijunedussci@126.com)

[1] School of Management, Hunan Institute of Engineering, Xiangtan, Hunan, China

[2] School of Clinical Medicine, Xiangtan Medicine and Health Vocational College, Xiangtan, Hunan, China

[3] School of business, Hunan Normal University, Changsha, Hunan, China

[#a] Current Address: Panlong Yuheyuan District, Yutang District, Xiangtan City, Hunan Province, China

[#b] Current Address: Hunan Normal University Erliban Campus, Yuelu District, Changsha City, Hunan Province, China

*Corresponding author

E-mail:

[¶]These authors contributed equally to this work.

[&]The author also contributed equally to this work.

Abstract

This study investigates the relationship between the educational level of village heads and the academic performance of adolescents, utilizing data from the China Family Tracking Survey (CFPS). The analysis reveals that village chiefs with higher education levels significantly enhance the academic outcomes of adolescents within their communities. This positive effect persists even when controlling for endogeneity through the use of instrumental variables and conducting a variety of robustness checks. Further investigation indicates that these educated village leaders contribute to an increased provision of public goods, thereby improving the external environment of the village which supports academic performance. Additionally, well-educated village chiefs serve as role models within the village's social network, influencing parental educational aspirations and thereby augmenting adolescents' academic results. Notably, the impact of highly educated village chiefs is more pronounced for girls and adolescents from low-income families, highlighting its significance for promoting gender equity in education and breaking cycles of intergenerational poverty.

Keywords: Village chiefs' educational attainment, public goods provision, role modeling effect, adolescent education

1. Introduction

The development of rural education is a crucial factor in driving rural economic growth, cultural prosperity, and achieving rural revitalization. As the foundation of the dynamic development process of rural education, the education of rural youth is key to the endogenous

drive for high-quality development in rural areas. Urbanization is a necessary process in social development, especially in developing countries, but it simultaneously causes the loss of rural elites and young labor forces (Liu et al., 2010)[1]. Currently, most developing countries face the dual dilemma of insufficient school teaching resources and lack of family education for many rural youths (Stelmach, 2011; Smitha, 2014; Du Plessis, 2014)[2-4]. Therefore, under the premise that it is difficult to immediately improve rural teacher teams and integrate urban and rural education, it is necessary to explore the factors affecting the development of rural youth education from a third-party field and seek ways to optimize rural human capital.

Existing literature discussing factors affecting the development of rural youth education primarily focuses on the roles of schools and family education (Byun et al., 2012; Corbett and Forsey, 2017; Lichter et al., 1993; McGrath, 2001; Yiu and Adams, 2013)[5-9]. As the main fields where youth receive education, schools and families have a direct impact on the initial accumulation of human capital. Additionally, some scholars have focused on the peer effect on rural children's education, showing that negative peer effects can reduce academic performance and increase dropout rates among adolescents (Chen, 2021; Li, 2013 ; Helmers and Patnam, 2014; Sacerdote, 2011)[10-13]. This line of research highlights that the external environment, alongside schools and family education, plays an equally important role in accumulating human capital for rural children. Besides schools and families, the external village environment, including public infrastructure, social networks, village security, and environmental pollution, constitutes another significant field. Theoretically, village public infrastructure is a crucial resource for youth learning and development; the characteristics of

village social networks may subtly influence the formation of youth values and worldviews; and village security and environmental pollution can also affect the cultivation of cognitive and non-cognitive abilities in youth. Therefore, if the external village environment indeed affects youth education, enhancing governance of this environment becomes crucial.

The village head is the main manager of the village environment, and their quality, capability, and educational level directly impact their governance ability. However, existing literature on village governance mainly discusses the impact of village leaders' quality, education, and authority on village economic development and household income, paying insufficient attention to governance of the external environment (Berthu et al., 2019; Jain et al., 2023; McManus and Hanna, 2014; Nadia and Mahi, 2023; Rozelle, 1998)[14-18]. Could a more educated village head improve the village environment? And would such improvement in the external environment benefit the educational development of village youth? Clearly, against the backdrop of rural elite loss and the demands of rural revitalization strategies, answering these questions could provide important policy references and practical bases for the selection mechanisms of rural grassroots cadres and rural education development.

Academic performance is an important indicator of educational development. Based on this, the paper utilizes data from the China Family Panel Studies (CFPS) to empirically test the impact of village heads' educational levels on the academic performance of adolescents and explores the internal mechanisms of how the education level of village heads affects youth academic performance from the perspective of the external village environment. Besides the aforementioned policy implications, this paper also makes marginal contributions:

firstly, it discusses rural youth education development from the perspective of the external village environment, finding that besides school and family environments, the village environment also significantly impacts youth education development, supplementing existing literature. Secondly, the paper integrates the education level of village heads, village environment, and rural youth education into a unified analytical framework, analyzing the intergenerational impact of village heads' education levels on youth education during the village governance process, enriching the theory of rural “elite governance”.

2. Literature Review and Theoretical Hypotheses

2.1. External Environment and Youth Educational Development

Beyond school education and family education, an increasing number of scholars have begun to focus on the impact of the external environment on the educational development of young people. It is widely believed that the time a person spends living in a specific community environment during childhood is a critical determinant of their long-term educational outcomes and economic performance (Brooks-Gunn et al., 1993; Wilson, 1987)[19, 20]. In recent years, scholars led by Chetty have begun to reevaluate the effects of neighborhood environments. Chetty and colleagues (2016; 2018a; 2018b) found that neighborhood environments not only significantly impact the education of minors but also demonstrated that improvements in the external environment during childhood can increase college admission rates, ultimately affecting intergenerational mobility[21-23]. Liu (2019) showed that compared to children in rural communities, those living in urban communities

have stronger language skills[24]. Wang et al (2020), using data from the China Family Education Survey, studied the impact of neighborhood environments on student performance, finding that a good neighborhood environment can significantly improve students' academic achievements[25].

Regarding the mechanisms through which external environments affect the educational development of adolescents, they can generally be divided into four aspects: First, community resource and facility disparities. Recreational facilities, kindergartens, primary schools, and other educational institutions have a significant impact on children's education within the community, and communities with fewer resources often lack high-quality community resources and facilities, thereby affecting the educational development of adolescents. Second, the impact of environmental pollution. Adolescents' long-term exposure to environmental pollution can affect their cognitive development (Power et al., 2016)[26], thereby reducing academic performance. Third, the social network relationships within the community are important factors affecting children's education. On one hand, communities with fewer resources lack role models for demonstration and guidance, which is not conducive to inspiring students to pursue success in school (Wang et al., 2020; Wilson, 1987)[20, 25]. On the other hand, harmonious neighborhood relationships strengthen social co-monitoring (Sampson et al., 1997)[27], and expand the family community network, which is beneficial for children's access to educational resources (Christenson and Sheridan, 2001)[28]. Fourth, the level of community security. Poor security levels cause parents to reduce the scope and duration of children's outdoor activities, limiting opportunities for children to interact with peers and adults (Furstenberg, 1993)[29]. Moreover, adolescents who suffer or witness

community violence may experience severe depression, anxiety, heightened vigilance, aggressive behavior, and worsened academic performance (Fowler et al., 2009; Sharkey et al., 2014)[30, 31].

2.2. Educational Level of Village Heads and Governance of Village Environments

Since the implementation of the village self-governance system, the governance model of village organizations has continued to exhibit the characteristics of rural elite governance, forming a diverse governance model under the village election autonomy model and village cadre selection mechanism, which includes "talented people governing the village," "wealthy people governing the village," "clan governance," and "village sages governing the village." Among these, "talented people governing the village" involves the election of village cadres with high quality, rich production experience, or high educational backgrounds through grassroots democratic systems to manage the village. These village cadres internalize their human capital into governance capabilities while guiding village production practices and implementing national policies, thereby playing an important role in rural economic development and income growth for rural households. For instance, Nadia (2023) demonstrated that the quality characteristics of village cadres are significantly related to village economic development[17]. Village cadres' cultural quality facilitates the acquisition of new knowledge, and their comprehensive ability quality possesses strong rural affairs handling capabilities, ultimately impacting rural economic development. Zhao and He (2016) studied the impact of village cadres' quality on villagers' income, finding that

high-quality village cadres can enhance villagers' income by attracting external investments and acquiring political resources[32]. More directly, Gao and Bi (2009), Liu and Mao (2015) examined the impact of village cadres' educational levels on rural household income and non-agricultural income, both finding a significant income growth effect associated with higher-educated village cadres[33, 34]. Yang and He (2019) further showed that village heads with higher educational backgrounds are beneficial in stimulating entrepreneurial vitality and enhancing property rights protection, significantly promoting village economic development[35].

In summary, both reality and empirical experience indicate that the knowledge and education of village leaders play an important role in promoting rural economic development, increasing agricultural output, and boosting farmers' income. However, despite the significant contributions of highly educated village cadres to village economic development, studies on whether highly educated village cadres can effectively provide public goods and improve the external village environment are relatively rare and subject to debate. Proponents argue that highly educated village cadres have strong control over the financing, operation, and maintenance of rural public goods supply, and they also possess robust social networks, thereby facilitating the acquisition of public product investments (Yao, 2011)[36]. Critics, however, argue that the rational individual assumption and the motive for personal benefit realization hinder village cadres from undertaking public duties. Furthermore, the disparity between the educational rate of return and actual income may stimulate village cadres' economic motives while weakening their willingness to provide public services. Ren et al. (2020) found that highly educated college student village officials are more focused on

economic development and neglect environmental protection[37]. Additionally, due to the national power extending to the countryside, the standardization and bureaucratization of village governance, managing trivial rural public affairs becomes challenging, which could also be a factor causing the public governance dysfunction of highly educated village cadres. Yang and He (2019) suggest that although highly educated village heads fully utilize internal and external resources to develop the village economy initially, in later stages, long-term village chiefs will focus on non-economic aspects such as village culture, civility, and cleanliness in the management and construction of the village[35].

Through the organization and review of existing literature, it is not difficult to see that the neighborhood environment is an important factor affecting the development of adolescents. However, this paper finds that in the existing literature on neighborhood environments and the educational growth of adolescents, few scholars have focused on the impact of the village environment on the development of adolescent education. In rural areas, where school education quality is often lacking and family education is frequently absent, discussing the impact of the rural community environment on adolescent education development has more practical significance compared to urban communities. At the same time, whether highly educated village leaders and other village-level managers are more conducive to promoting the governance of the external village environment remains to be further investigated. This paper argues that the existing literature on the governance of the external village environment mostly starts from the perspectives of village cadres and college student village officials. However, village cadres lack decision-making power in the acquisition and allocation of collective resources, and college student village officials not

only find it difficult to grasp the formal power resources of the village but also have high mobility, making it difficult for them to be deeply rooted in rural governance for a long time (Hu, 2019)[38]. The nature of duties and powers of different entities might be the main reasons for the lack of consensus. The village head is one of the cores of village governance and has considerable decision-making power in the management of village affairs. Generally, the village head is a local villager with a strong local attachment and a stronger willingness to participate in village environmental governance (Wang et al., 2020)[39].

2.3. Theoretical Hypotheses

Theoretically, the educational level of village heads may affect the educational development of adolescents through the following pathways: First, education not only makes individuals value educational investment but also enhances their altruism. Therefore, highly educated village heads are likely to show greater intergenerational concern, valuing the educational development of the village's next generation and directly influencing adolescent education. Second, family income is an important factor affecting adolescent education. Existing literature shows that highly educated village heads can effectively promote village economic development and family income growth. Consequently, highly educated village heads can reduce the rate of parents working away from home and increase their willingness to return, indirectly affecting home education. Additionally, they can influence adolescent education through increased family income, by enhancing the material resources and opportunities available for education.

Third, highly educated village heads can use their knowledge, abilities, and local

attachments to obtain public product resources externally and expand public product supply internally, thus promoting the development of an ecologically livable and culturally advanced external village environment. Therefore, they can influence adolescent education development by improving the external environment of the village through the provision of public goods. Finally, the significant economic effects displayed by highly educated village heads in using knowledge and technology to promote rural economic development and increase villagers' income can enhance parents' expected returns from education, thus affecting the emphasis parents place on their children's education. Thus, highly educated village heads have a role model effect in the social network relationships of the external village environment. This role model effect is further enhanced in the guidance of village production practices and in the daily interactions of village life. It is important to note that while the income growth effect is one pathway through which the educational level of village heads affects adolescent educational development, the last two pathways—public goods supply and role model effect—are the main focus of this paper.

Based on this, the paper proposes the following hypotheses:

Hypothesis 1: The educational level of village heads will significantly affect the academic performance of adolescents. This implies that highly educated village heads will improve the academic performance of adolescents.

Hypothesis 2: The educational level of village heads will affect adolescent academic performance through their impact on the external village environment. Specifically, highly educated village heads will promote adolescent academic performance by increasing the supply of public goods and through their own role model effect.

3. Research design

3.1. Data Sources

The data used in this paper come from the China Family Panel Studies (CFPS) by the Institute of Social Science Survey at Peking University. CFPS is a nationwide, large-scale, multidisciplinary social tracking survey project that covers 25 provinces, cities, and autonomous regions in China, capturing a wide range of information, including family economic activities, educational outcomes, family relationships, family dynamics, population migration, and characteristics of the communities/villages where families reside. To date, CFPS has made public the data from 2010, 2012, 2014, 2016, 2018, and 2020, but only the 2010 and 2014 datasets include community/village-level data. Thus, according to the research objectives, this paper selects the 2014 CFPS data for empirical analysis. The 2014 CFPS data include basic characteristics of 408 villages and information on academic performance of adolescents within these villages. After retaining rural samples, adolescent samples, and removing missing values for various variables, a total of 1,720 valid samples were retained.

3.2. Variable Selection

3.2.1. Educational Level of Village Heads

The educational level of village heads is the main explanatory variable in this paper, measured using the Educational Level of Village/Community Heads from the CFPS community/village questionnaire. This variable is ordinal, with values ranging from 1 to 6, corresponding to illiterate/semi-literate, primary school, middle school, high school, junior

college, and undergraduate or higher education levels.

3.2.2. Academic Performance of Adolescents

In existing research, scholars often use exam scores or standardized scores to measure the academic performance of adolescents. However, due to variations in exam difficulty and scoring standards across different regions, these scores are not an appropriate variable to measure academic performance. This paper uses the average scores of the word group test and mathematics test from the child cognitive module in CFPS data to measure the academic performance of adolescents. The advantages of this approach include the uniformity of the word group test and mathematics test in CFPS data, which ensures the same level of difficulty, and the objective nature of the test questions, which ensures consistent scoring standards. However, more educated children might achieve higher scores, so this paper standardizes the test scores according to the children's current educational level to reduce intergroup differences. Additionally, an age variable for the children is added in the econometric model to control as much as possible for the educational and age trends in adolescent academic performance. It is worth noting that to ensure the scientific validity and comparability of test scores, the CFPS team only tests individuals aged 10 years and older; the CFPS children's questionnaire only includes children under the age of 16. Therefore, the sample interval for adolescents in this paper is 10-15 years old. It is worth noting that standardization is performed under the original sample of rural families without excluding missing variables. Therefore, the standard deviation of the Academic performance variable in empirical analysis is not 0.

3.2.3. Control Variables

Drawing on existing literature, this paper controls for the gender of the village head (male = 1; female = 0). On one hand, the gender of the village head may influence preferences for economic investment and public services; on the other hand, female village heads may exhibit more intergenerational care during village governance. The age of the adolescent is an important factor affecting performance on the CFPS academic tests; therefore, it is controlled for. Additionally, as adolescents are influenced by external environments with variations by age and gender, the gender of the adolescent is also controlled. At the parental level, parents' education directly influences adolescent academic performance, and parents' political status can provide better educational resources through political capital. Therefore, this paper controls for the highest level of education of parents (values ranging from 1 to 6, similar to the educational level of the village head) and the highest political status (party member = 1; otherwise = 0).

In terms of family characteristics, family income is an important factor affecting adolescents' access to educational resources. In rural areas, the insurance and income-increasing effects of land assets influence family education decisions. Furthermore, the income risk associated with agricultural production restricts education investments, and non-farm employment involving labor migration can adversely affect left-behind adolescents. Existing studies have shown that the internet can also impact the quantity and quality of adolescents' education and the accumulation of new human capital. The number of family health shocks and the number of working family members can respectively enhance and weaken family budget constraints. Increases in the number of health shocks and working

family members can affect parental involvement and family education investment, thereby impacting adolescent academic performance. Therefore, this paper also controls for family characteristics such as family income (logarithmic), land assets (logarithmic), nature of family production (agricultural production = 1; otherwise = 0), internet access at home (yes = 1; no = 0), the number of family health shocks, and the number of working family members. Descriptive statistics of the variables are shown in Table 1.

Table 1. Descriptive Statistics of Variables

Variable	Observations	Mean	S.D	Min	Max
Academic performance	1 720	-0.12	0.90	-2.54	2.21
Educational Level of Village Heads	1 720	3.56	0.91	1	6
Gender of Village Director	1 720	0.98	0.151	0	1
Teenage age	1 720	12.42	1.737	10	15
Teenage Gender	1 720	0.52	0.5	0	1
The highest educational level of parents	1 720	2.16	1.03	1	6
Political identity of parents	1 720	0.10	0.31	0	1
Household income (log)	1 720	10.18	1.22	0.69	14.12
Land assets (log)	1 720	8.29	4.05	0	14.27
Agricultural production	1 720	0.82	0.39	0	1
Internet	1 720	0.36	0.48	0	1
No. Health shock	1 720	0.51	0.75	0	5
No. Household workers	1 720	2.18	1.09	0	8

3.3. Econometric models

According to the research purpose of this article, the following benchmark econometric model is set:

$$Edu\_Score_i = \alpha_1 + \beta_2 Edu\_year_i + \beta_3 Control_i + \varepsilon_i \quad (1)$$

Among them, Edu_Score represents the average of the youth phrase test and math test, Edu_year represents the education level of the village head, $Control$ represents various control variables at the individual characteristics, youth characteristics, and family level of the village head, and ε represents the random error term.

4. Empirical Analysis and Results

4.1. Educational Level of Village Heads and Adolescent Academic Performance

Table 2 reports the Ordinary Least Squares (OLS) regression results on the impact of the educational level of village heads on adolescent academic performance. Column (1) does not control for any variables and simply analyzes the relationship between the educational level of village heads and adolescent academic performance. It is evident that the educational level of village heads is significantly positively correlated at the 1% level with the adolescent's academic performance, with a coefficient of 0.120. This means that for every unit increase in the educational level of the village head, the academic performance score of adolescents in the village increases by 0.12 standard deviations.

To further reduce the impact of omitted variables and increase the robustness of the model, Columns (2) and (3) progressively include individual characteristics of adolescents, individual characteristics of parents, and family characteristics. The results in Column (2) indicate that even after controlling for individual characteristics of adolescents and parents, which significantly impact academic performance, the educational level of village heads still shows a significant positive correlation with adolescent academic performance, although the effect size decreases. After further controlling for family characteristics in Column (3), the coefficient decreases to 0.096, but the educational level of the village head remains significantly positively correlated with adolescent academic performance at the 1% level. For every unit increase in the educational level of the village head, the standard deviation of

adolescent academic performance scores increases by 0.096.

Therefore, the results of the stepwise regression indicate that the educational level of village heads is an important factor affecting adolescent academic performance. Human capital is a crucial element for village construction and economic development. With the increasing proportion of rural labor migration, the phenomenon of left-behind children has intensified, and ensuring the sustainable economic development of rural areas is key to rural revitalization. The findings of this paper imply that as the core of the village elite class, village heads with higher education not only play a vital role in current rural production and economic development but also in the cultivation of human capital for the next generation.

Table 2. Education level of village directors and academic performance of adolescents

Variable	(1)	(2)	(3)
	Academic performance		
Edu_year	0.120***	0.106***	0.096***
	(0.023)	(0.021)	(0.021)
Gender of Village Director		0.019	0.018
		(0.025)	(0.024)
Teenage age		0.228***	0.228***
		(0.012)	(0.011)
Teenage Gender		-0.093**	-0.086**
		(0.038)	(0.038)
Highest_edu of parents		0.133***	0.106***
		(0.019)	(0.019)
Party_parents		-0.106*	-0.096
		(0.063)	(0.064)
Household income			0.054***
			(0.016)
Land assets			-0.011
			(0.007)
Agricultural production			0.036
			(0.080)
Internet			0.167***
			(0.043)
No. Health shock			-0.022
			(0.029)
No. Household workers			-0.044**
			(0.022)
Observations	1 720	1 720	1 720

Note: The robust standard error is indicated in parentheses; ***, **, and * represent significant at the 1%, 5%, and 10% levels, respectively.

4.2. Addressing Endogeneity Issues

The sources of endogeneity primarily include reverse causality and omitted variables. Although the academic performance of adolescents does not affect the educational level of village heads, thereby eliminating the possibility of reverse causality, omitted variables remain a significant factor causing endogeneity in the baseline model. Therefore, this paper attempts to mitigate endogeneity issues using the instrumental variable (IV) approach. The paper employs the average educational level of village heads at the county level as the instrumental variable. On one hand, policy-wise, within the same area, the arrangement of village heads across different villages has a certain policy-relatedness (Yang and He, 2019); in democratic elections, the election culture and preferences within the same county are similar, thus exhibiting correlations in the choice between governance modes such as clan governance, wealthy governance, or village sage governance, fulfilling the relevance requirement for the instrumental variable. On the other hand, the average educational level of village heads in a county does not directly impact the academic performance of adolescents within a single village, satisfying the exclusivity of the instrumental variable. However, due to the CFPS data possibly having fewer samples for some counties' villages, this could lead to a weak instrumental variable problem. Therefore, following existing literature, the paper also relaxes the level of the instrumental variable to the provincial level, using the average educational level of village heads at the provincial level (Gao and Bi, 2009; Yang and He, 2019).

Table 3 reports the Two-Stage Least Squares (2SLS) regression results. Columns (1) and (2) report the first-stage regression results using the average educational level of village heads at the county and provincial levels as instrumental variables, respectively. The results show

that both instrumental variables are significantly positively correlated with the educational level of village heads, confirming the relevance of the instrumental variables. At the same time, the results for weak instrumental variable tests at the bottom of Columns (1) and (2), with the C-D Wald F-statistics significantly exceeding 16.38, exclude the possibility of weak instrumental variables. Columns (3) and (4) report the second-stage regression results of 2SLS. It is evident that, whether using the average educational level of village heads at the county or provincial level as the instrumental variable, the educational level of village heads is significantly positively correlated with adolescent academic performance at the 1% level. Therefore, using instrumental variables confirms that the educational level of village heads has a positive effect on the academic performance of adolescents.

Table 3. Endogeneity problem handling

Variable	(1)	(2)	(3)	(4)
	First stage		Second stage	
	Edu_year		Academic performance	
Edu_year			0.177***	0.223***
			(0.030)	(0.063)
IV1	0.974***			
	(0.028)			
IV2		0.879***		
		(0.077)		
C-D Wald Fstatistic	1688.73	202.169		
Observations	1 720	1 720	1 720	1 720

Note: The robust standard error is indicated in parentheses; ***, **, and * represent significant at the 1%, 5%, and 10% levels, respectively.

4.3. Robustness Tests

To further affirm the conclusion that the educational level of village heads impacts adolescents' academic performance, this paper conducts robustness tests from the following aspects:

4.3.1. Redefining the Educational Level of Village Heads

The educational level of village heads was redefined into three binary dummy variables: university degree, high school diploma, and middle school education or below. The regression results reported in Table 4, Columns (1) to (3), show that village heads with a university degree and high school education have a significantly positive impact on adolescents' academic performance. The coefficient for university education is 0.131, higher than that for high school education at 0.073, indicating that the higher the education, the greater the positive impact on adolescents' academic performance. Notably, village heads with education of middle school or below no longer have a positive effect on adolescents' academic performance and may instead hinder their academic improvement. These results confirm the robustness of the baseline results and highlight the importance of cultivating high-quality village management personnel in the accumulation of rural human capital.

4.3.2. Replacing the Variable for Adolescent Academic Performance

The paper uses parents' subjective perceptions of their adolescents' performance in language and mathematics as measures of academic performance. This categorical variable ranges from 1 to 4, representing excellent, good, average, and poor, respectively. Results in Table 4, Columns (4) and (5), clearly show that the educational level of village heads significantly affects parents' evaluations of adolescents' performance; higher educational levels of village heads correlate with more positive parental evaluations, indicating that the baseline result regarding the impact of village heads' educational level on adolescent academic performance is robust.

4.3.3. Considering Changes in Village Heads

Given the term limits and election fluctuations for village heads, changes in office may cause fluctuations in their educational levels. If such changes occur within a short period, they could bias the estimated results. Therefore, using CFPS data from 2010, the paper re-estimates Equation (1) after retaining samples where the educational levels of village heads remained unchanged between 2010 and 2014. The results in Column (6) indicate that even after accounting for changes in village heads, their educational level significantly impacts adolescents' academic performance at the 1% level.

4.3.4. Adjusting Clustered Standard Errors

Considering that adolescents' academic performances are correlated at the village level, the paper further clusters standard errors at the village level to allow for any intra-village correlation. The results after adjusting for clustered standard errors, as shown in Column (7), remain robust.

Table 4. Robustness Test

Variable	(1)	(2)	(3)	(4)	(5)	(6)	(7)
	Academic performance			language	mathematics	Academic performance	
Edu_year				-0.024*	-0.037**	0.159***	0.096***
				(0.014)	(0.016)	(0.042)	(0.036)
University degree	0.131***						
	(0.048)						
High school education		0.073*					
		(0.038)					
Junior high school and below			-0.129***				
			(0.038)				
Control variables	YES	YES	YES	YES	YES	YES	YES
Observations	1 720	1 720	1 720	2 868	2 871	624	1 720

Note: The robust standard error is indicated in parentheses; ***, **, and * represent significant at the 1%, 5%, and 10% levels, respectively.

4.4. Mechanism Analysis

After addressing endogeneity issues and conducting multiple robustness checks, the previous sections have confirmed that the educational level of village heads is a significant factor influencing adolescent academic performance. The next question is: through what specific mechanisms does the educational level of village heads affect adolescent academic performance? This section will further validate Hypothesis 2 using the three-step method of mechanism testing known as the BK (Baron and Kenny) method.

Step One involves examining the impact of the educational level of village heads on adolescent academic performance, as specified in Equation (2).

Step Two assesses whether there is a significant impact of the educational level of village heads on the mechanism variables, as specified in Equation (3).

Step Three incorporates both the educational level of village heads and the mechanism variables into the model, as shown in Equation (4).

If, in Equation (3), the mechanism variable is significant and the coefficient of the village head's educational level decreases, then the mechanism is considered valid. The equations are laid out as follows:

$$Edu\_Score_i = \alpha_1 + \beta_2 Edu\_year_i + \beta_3 Control_i + \varepsilon_i \quad (2)$$

$$Mech_i = \alpha_1 + \beta_2 Edu\_year_i + \beta_3 Control_i + \varepsilon_i \quad (3)$$

$$Edu\_Score_i = \alpha_1 + \beta_2 Edu\_year_i + \beta_3 Mech_i + \beta_4 Control_i + \varepsilon_i \quad (4)$$

This structured approach allows for a clear assessment of whether the proposed mechanisms (such as increased access to educational resources, improved public goods, or role model effects) mediate the relationship between the educational level of village heads

and adolescent academic performance. By examining the significance and the size of the coefficients in these equations, one can determine the extent to which these mechanisms play a role in influencing adolescent outcomes.

4.4.1. Public Goods Supply

This paper uses the expenditure on public services within the village to measure the supply of public goods, which is treated as a continuous variable and log-transformed. Additionally, a poor and disorganized living environment can impact adolescents' cognitive abilities to some extent and can negatively model behavior, thus affecting academic performance. Therefore, this section further discusses whether highly educated village heads can enhance adolescent academic performance through the management of environmental pollution in village living environments. The cleanliness of village roads is used as a proxy variable for the management of the village living environment. This is a categorical variable with values ranging from 1 to 7, where higher values indicate cleaner roads. Table 5 reports the regression results for this part. Columns (1) and (2) re-estimate the impact of the village head's educational level on adolescent academic performance after accounting for missing values in the mechanism variables. Columns (3) and (4) report the impact of the village head's educational level on the supply of public goods and environmental pollution management, showing that the educational level of village heads significantly affects the mechanism variables. The results in Columns (5) and (6) indicate that both the supply of public goods and environmental pollution management in the village affect adolescent academic performance, and the coefficient of the village head's educational level is lower compared to Columns (1)

and (2). This suggests that enhancing the supply of public goods is an important mechanism through which the educational level of village heads influences adolescent academic performance.

Table 5. Mechanism Analysis of Village Residential Environment Governance

Variable	(1)	(2)	(3)	(4)	(5)	(6)
	Academic performance		Public goods supply	Pollution control	Academic performance	
Edu_year	0.111***	0.096***	0.223***	0.146***	0.105***	0.092***
	(0.023)	(0.021)	(0.051)	(0.041)	(0.024)	(0.021)
Public goods supply					0.029**	
					(0.012)	
Pollution control						0.030**
						(0.014)
Control variables	YES	YES	YES	YES	YES	YES
Observations	1 291	1 720	1 291	1 720	1 291	1 720

Note: The robust standard error is indicated in parentheses; ***, **, and * represent significant at the 1%, 5%, and 10% levels, respectively.

4.4.2. Role Model Effect

The role model effect's inherent manifestation in education is the motivation and expectation for education. Referring to the approach of Wang et al. (2020), this paper uses parents' educational expectations for adolescents to measure the role model effect. Educational expectations are measured using the CFPS question "Desired Level of Education for Children," with values ranging from 1 to 8, corresponding to no schooling necessary, primary school, middle school, high school, junior college, bachelor's degree, master's degree, and doctorate. Columns (1) to (3) of Table 6 report the results of the tests for the role model effect mechanism. These columns correspond to the first, second, and third steps of the three-step method, respectively. It can be seen that the educational level of village heads significantly influences parents' educational expectations for their children. The higher the educational level of the village head, the greater the parents' educational expectations,

indicating that highly educated village heads exert a role model effect. The educational expectations are significantly positively correlated with adolescent academic performance at the 1% level in Column (3), further confirming that this role model effect can effectively enhance adolescent academic performance, with a noticeable decrease in the coefficient of the village head's educational level compared to Column (1). These results indicate that the role model effect is an important mechanism through which the educational level of village heads influences adolescent academic performance.

Table 6. Mechanism Analysis of Example Effect

Variable	(1)	(2)	(3)
	Academic performance	Educational expectations	Academic performance
Edu_year	0.098***	0.101***	0.085***
	(0.021)	(0.031)	(0.020)
Educational expectations			0.129***
			(0.017)
Control variables	YES	YES	YES
Observations	1 712	1 712	1 712

Note: The robust standard error is indicated in parentheses; ***, **, and * represent significant at the 1%, 5%, and 10% levels, respectively.

4.5. Heterogeneity Analysis—Adolescent Gender and Family Income

The discussions above regarding the impact of village heads' educational levels on adolescent academic performance are based on average implications. However, the effect may vary across different groups. This section will conduct a heterogeneity analysis focusing on the gender of adolescents and family income.

Columns (1) and (2) of Table 7 report the regression results segmented by adolescent gender. The results show that compared to boys, girls are more influenced by the educational level of village heads. The mechanism analysis in this paper finds that highly educated village heads can improve the village living environment by increasing the supply of public goods. Existing research indicates that improvements in living environments have a greater positive

impact on female adolescents than on male adolescents. This result is consistent with existing studies.

Columns (3) to (5) present model estimation results after dividing the sample into low-income families, middle-income families, and high-income families based on the 25th and 75th percentiles of income. For low-income families, the educational level of village heads significantly influences adolescent academic performance at the 1% level, with a coefficient of 0.160. For high-income families, highly educated village heads also significantly improve adolescent academic performance, with a coefficient of 0.084, which is lower than that for low-income families. In middle-income families, there is no significant correlation. Existing research suggests that highly educated village heads are beneficial for village economic development, promoting income growth for rural households and reducing poverty. This income growth effect has a greater impact on low-income families, allowing them to invest more resources in their children's education. Moreover, in social interactions and production processes, low-income families are more likely to be influenced by the role model effect of highly educated village heads, thereby increasing the likelihood of investing in education. The reason the educational level of village heads impacts the academic performance of adolescents from high-income families may be due to the complementary advantages of family educational resources and improved external environments enhancing the academic performance of adolescents from these families.

5. Conclusion and Policy Recommendations

5.1. Conclusion

Rural education development plays a crucial role in promoting income growth for rural households, consolidating poverty alleviation, and enhancing rural prosperity. As the core of the village elite class, the educational level of village heads not only directly influences rural economic development and social governance, but also has a positive intergenerational impact on the accumulation of human capital during village governance. This is key for the sustainable development of rural education in the context of rural revitalization. Based on the 2014 CFPS data, this paper studies the impact of the educational level of village heads on the academic performance of rural adolescents and the underlying mechanisms. It was found that highly educated village heads significantly improve children's academic performance. This result holds even after addressing endogeneity issues and applying various robustness tests. The mechanisms include: (1) highly educated village heads focusing more on the supply of public goods, thereby improving the external environment of the village, which affects adolescent academic performance; (2) highly educated village heads having a significant role model effect on the parents of adolescents, influencing their educational expectations and thereby impacting academic performance. Further heterogeneity analysis found that highly educated village heads have a greater positive impact on the academic performance of girls and children from low-income families.

5.2. Policy Recommendations

Talent Retention in Rural Governance: As urbanization progresses, there is a substantial migration of rural intellectual elites to cities, leading to a talent shortage in village governance. This study shows that highly educated village heads have a positive intergenerational impact on human capital accumulation, and the continued loss of these elites not only hinders current rural economic development but also weakens the driving force for sustainable rural economic development. Thus, it is crucial to improve the compensation and incentive systems for village-level management personnel to attract and retain more highly educated individuals in village governance. Additionally, enhancing the selection mechanisms for village cadres and ensuring robust rural democratic elections can help capable, highly educated individuals lead through competitive merit-based systems.

Role Model Effect of College-Educated Village Officers: This conclusion provides empirical support for the existing College-Educated Village Officer system. These officers, when embedded in rural governance, significantly influence local parents and encourage investment in their children's education and motivate adolescents to learn.

Improving Public Services: Highly educated village heads can enhance adolescents' learning and growth environments by improving public service provision, thus boosting academic performance. Therefore, both political elites within the village and embedded college-educated officers should work towards reforming village governance performance assessment systems, emphasizing the construction of public infrastructure and the improvement of living environments.

Enhancing Rural Living Conditions: Considering that the village living environment is a

crucial factor affecting adolescent educational development, governments should not only promote village self-governance but also actively advance rural construction initiatives, improve rural waste collection and disposal systems, and enhance the management of residential environment facilities, increasing efforts to combat rural crime and promoting the construction of safe rural communities.

Declaration of conflicting interests

The author(s) declared no potential conflicts of interest with respect to the research, authorship, and/or publication of this article.

Availability of data and code

The data and codes presented in this study are available upon request from the corresponding author.

Funding

Not applicable.

References:

1. Liu Y, Liu Y, Chen Y, Long H. The process and driving forces of rural hollowing in China under rapid urbanization. J Geogr Sci. 2010;20(6):876-88. http://doi.org/10.1007/s11442-010-0817-2
2. Stelmach BL. A Synthesis of International Rural Education Issues and Response. Rural Educator.

2011;32(2):32-41.
3. Smitha A, University OU. Contemporary Challenges for Education in Conflict Affected Countries. Journal of international and comparative education. 2014;3(1):113-25. http://doi.org/10.14425/00.62.86
4. Du Plessis P. Problems and complexities in rural schools: Challenges of education and social development. Mediterranean Journal of Social Sciences. 2014;5(20):1109-17.
5. Byun S, Meece JL, Irvin MJ, Hutchins BC. The Role of Social Capital in Educational Aspirations of Rural Youth. Rural Sociol. 2012;77(3):355-79. http://doi.org/10.1111/j.1549-0831.2012.00086.x
6. Corbett M, Forsey M. Rural youth out-migration and education: challenges to aspirations discourse in mobile modernity. Discourse (Abingdon, England). 2017;38(3):429-44. http://doi.org/10.1080/01596306.2017.1308456
7. Lichter DT, Cornwell GT, Eggebeen DJ. Harvesting Human Capital: Family Structure and Education Among Rural Youth 1. Rural Sociol. 1993;58(1):53-75. http://doi.org/10.1111/j.1549-0831.1993.tb00482.x
8. McGrath B. “A problem of resources”: defining rural youth encounters in education, work & housing. J Rural Stud. 2001;17(4):481-95. http://doi.org/10.1016/S0743-0167(01)00023-7
9. Yiu L, Adams J. Reforming Rural Education in China: Understanding Teacher Expectations for Rural Youth. The China quarterly (London). 2013;216(216):993-1017. http://doi.org/10.1017/S0305741013001136
10. Chen Q. Why am I late for school? Peer effects on delayed school entry in rural northwestern China. Education economics. 2021;29(6):624-50. http://doi.org/10.1080/09645292.2021.1926932
11. Li Q, Zang W, An L. Peer effects and school dropout in rural China. China Econ Rev. 2013;27:238-48. http://doi.org/10.1016/j.chieco.2013.04.002
12. Helmers C, Patnam M. Does the rotten child spoil his companion? Spatial peer effects among children in rural India. Quant Econ. 2014;5(1):67-121. http://doi.org/10.3982/QE192
13. Sacerdote B. Peer effects in education: How might they work, how big are they and how much do we know thus far? Handbook of the Economics of Education: Elsevier; 2011. p. 249-77.
14. Berthu Y, Galib S, Jamaluddi J. The role of the village chief in governance of Siron Olong Village, Kalimantan Province, Indonesia. European Journal of Political Science Studies. 2019;3(1).
15. Jain C, Kashyap S, Lahoti R, Sahoo S. The impact of educated leaders on economic development: Evidence from India. J Comp Econ. 2023;51(3):1068-93. http://doi.org/10.1016/j.jce.2023.05.001
16. McManus J, Hanna R. Educational qualifications of village leaders in north india: Implications for public service delivery. John F.: Kennedy School of Government; 2014.
17. Nadia A, Mahi BR. Village Development: Effect of Vilage Fund and Village Head Education. Economics Development Analysis Journal. 2023;12(2):141-56. http://doi.org/10.15294/edaj.v12i2.66675
18. Rozelle S, Li G. Village leaders and land-rights formation in China. The American economic review. 1998;88(2):433-8.
19. Brooks-Gunn J, Duncan GJ, Klebanov PK, Sealand N. Do Neighborhoods Influence Child and Adolescent Development? The American journal of sociology. 1993;99(2):353-95. http://doi.org/10.1086/230268
20. Wilson WJ. The truly disadvantaged: the inner city, the underclass, and public policy. Chicago: University of Chicago Press; 1987.
21. Chetty R, Hendren N, Katz LF. The effects of exposure to better neighborhoods on children: New

evidence from the moving to opportunity experiment. The American economic review. 2016;106(4):855-902. http://doi.org/10.1257/aer.20150572
22. Chetty R, Hendren N. THE IMPACTS OF NEIGHBORHOODS ON INTERGENERATIONAL MOBILITY I: CHILDHOOD EXPOSURE EFFECTS. The Quarterly journal of economics. 2018;133(3):1107-62. http://doi.org/10.1093/qje/qjy007
23. Chetty R, Hendren N. THE IMPACTS OF NEIGHBORHOODS ON INTERGENERATIONAL MOBILITY II: COUNTY-LEVEL ESTIMATES. The Quarterly journal of economics. 2018;133(3):1163-228. http://doi.org/10.1093/qje/qjy006
24. Liu T. Is the “Three Moves of Mencius' Mother” Really Necessary? --An analysis of the impact of community environment on children's academic achievement. Beijing Social Science. 2019(1):87-98.
25. Wang J, Zhang K, Lu Y. Close to Zhu, the red:Neighborhood environment and students' academic performance. Economics (Quarterly). 2020;19(2):521-44.
26. Power MC, Adar SD, Yanosky JD, Weuve J. Exposure to air pollution as a potential contributor to cognitive function, cognitive decline, brain imaging, and dementia: A systematic review of epidemiologic research. Neurotoxicology (Park Forest South). 2016;56:235-53. http://doi.org/10.1016/j.neuro.2016.06.004
27. Sampson R, Raudenbush S, Earls F. Neighborhoods and Violent Crime: A Multilevel Study of Collective Efficacy. Science (American Association for the Advancement of Science). 1997;277(5328):918-24.
28. Christenson SL, Sheridan SM. Schools and Families: Creating Essential Connections for Learning. The Guilford School Practitioner Series.: Guilford Publications, Inc; 2001.
29. Furstenberg. How families manage risk and opportunity in dangerous neighborhoods. Sociology and the public agenda. 1993:231-58.
30. Fowler PJ, Tompsett CJ, Braciszewski JM, Jacques-Tiura AJ, Baltes BB. Community violence: A meta-analysis on the effect of exposure and mental health outcomes of children and adolescents. Dev Psychopathol. 2009;21(1):227-59. http://doi.org/10.1017/S0954579409000145
31. Sharkey P, Schwartz AE, Ellen IG, Lacoe J. High Stakes in the Classroom, High Stakes on the Street: The Effects of Community Violence on Student’s Standardized Test Performance. Sociological science. 2014;1(14):199-220. http://doi.org/10.15195/v1.a14
32. Zhao R, He A. Village Cadre Quality, Grassroots Democracy and Farmers' Income-An Empirical Study Based on CHIPS. Nankai Economic Research. 2016(2):129-52.
33. Gao M, Bi L. The impact of knowledge and youthfulness of village cadres on farm household income: an empirical analysis based on micro panel data. Management World. 2009(7):77-84.
34. Liu H, Mao M. The impact of village leaders' education level on rural residents' non-farm income--an empirical analysis based on micro data. China Rural Economy. 2015;9:69-79.
35. Yang C, He X. Village chief authority and village development--An analysis of data based on a survey of 1,000 villages in China. Management World. 2019;35(4):90-109.
36. Yao S, Zhang S, Jiang H. Analysis of factors affecting rural public goods supply in grain producing areas - based on survey data from Anhui Province. Agricultural Technology and Economics. 2011(2):110-6.
37. Ren T, Yang X, Kang P. How can “college student village officials” serve rural revitalization? --An empirical study based on 10,700 village-level data from the third agricultural census. China Youth Research. 2020(11):29-52.

38. Hu Y. Survival Worries and Development Confusions: Role Reconstruction of Young Village Cadres under the Perspective of Rural Revitalization. China Youth Research. 2019(9):18-24.
39. Wang X, Zhang J. Tong Qingmeng. Does Place Attachment Help Increase Farmers' Willingness to Participate in Village Environmental Governance? --An analysis based on survey data from Hubei Province. China Population-Resources and Environment. 2020;30(4):136-48.
