# Supplementary material for: The impact of village heads’ educational levels on adolescent academic performance: Evidence from rural China
Source: PLoS One. 2025 Jan 2;20(1):e0315822. doi: 10.1371/journal.pone.0315822 (PMC11694985; doi:10.1371/journal.pone.0315822)
Supplement: Attachment — Submitted filename: Response to reviewers.docx [file pone.0315822.s002.docx]

Dear Editor and Reviewers,

Thanks very much for taking your time to review this manuscript. I really appreciate all your comments and suggestions! Please find my itemized responses in below and my revisions/corrections in the re-submitted files.

We hope this revised manuscript has addressed your concerns, and look forward to hearing from you.

We have uploaded a "Revised Manuscript with Track Changes." However, we apologize for not using the track changes feature during the earlier revisions. Instead, we have utilized the comments function to explain the previously revised sections.

Aside from the revisions mentioned below, we have made the following changes: 1. The surname of the second author was corrected from 'Deng Huan' to 'Huan Deng'; 2. During the revision stage, we received funding support, and we have added this information in the funding section.

Thanks again!

**Editor：**

| **Comments to the Author** | **Response and Revison** |
| --- | --- |
| When submitting your revision, we need you to address these additional requirements. | Dear Editor:  Many thanks for you insights and constructive comments. We hope the revisions we made have improved the paper and were able to address your concerns. The changes are highlighted in **red** in the manuscript file. Thank you ! |
| **Additional Comments**   1. Please ensure that your manuscript meets PLOS ONE's style requirements, including those for file naming. 2. Please include your full ethics statement in the ‘Methods’ section of your manuscript file. In your statement, please include the full name of the IRB or ethics committee who approved or waived your study, as well as whether or not you obtained informed written or verbal consent. If consent was waived for your study, please include this information in your statement as well. 3. Please include a copy of Table 7 which you refer to in your text on page 33 in PDF submission. | 1. Dear Editor, we have revised the manuscript according to the PLOS ONE style templates you provided. 2. The complete ethics statement has been added to the "Methods" section, specifically in lines 149-155. 3. We sincerely apologize for the oversight; Table 7 has been added on line 403. |

**Reviewer 1：**

| **Comments to the Author** | **Response and Revison** |
| --- | --- |
| Generally, this manuscript though well intended, is seemingly deficient in several areas. | Dear Reviewer 1:  Many thanks for you insights and constructive comments. We hope the revisions we made have improved the paper and were able to address your concerns. The changes are highlighted in **red** in the manuscript file. Thank you ! |
| **Introduction**   1. The manuscript is not clear in stating the economic or social platform that is used for information transfer or knowledge sharing by village elders to young persons in the population. 2. Assertions and suppositions, if not opinions, ought to be supported by appropriate citations/references/literature. This sadly was missing on several instances throughout the manuscript. 3. The author needs to give readers a better understanding of certain characteristics of the study population. What does rural mean in the context of the study? What does knowledge of the village elders speak to? Do they have university degrees etc.? what is it about them that is distinct and lends to the arguments being made? 4. The author needs to establish coherence. There are several instances where thoughts are disjointed and appear out of place. This is very much noticeable in the Sections 1 and 2 of the manuscript. The author would be best advised to make use of the funnel approach when constructing section 1/or revising same. 5. Additionally, this section lacks reference to any statistical/data to support some of the assertions made. One is not sure of the origin/source of certain statements. 6. The author is best advised to cite references when making statements and/or inferences that are not original to the author. Evidence of this is seen in the sentence in paragraph 2 of the 'Introduction' wherein there is a sentence that begins with 'therefore...' 7. In the final paragraph of the 'Introduction' the author introduced "besides the aforementioned policy implications..." This note in the opinion of the reviewer is best suited for the discussions or recommendations sections of the manuscript. Seeking to discuss policy implications at this juncture of the manuscript appears out of place. Additionally, the final sentence of the las paragraph of the "introduction" appears best suited for the section on 'research design/methodology' This sentence also appears to be out of place. The author is best advised to correct this. 8. Section 2 (Literature Review) is also replete with the use of inconsistent citation styles. In paragraph 2 of Section 2, the author attempts to introduce some four aspects of educational development of adolescents. It is apparent that the author made use of the incorrect punctuation mark after stating each of the four aspects. This made reading this section a bit of a challenge given repetitious use of words heretofore mentioned in the assumed heading sentences.(section 2.1). Additionally, having each of the four aspects in the same paragraph suggest that they are invariably connected when this is not necessarily the case. The author is advised to rephrase this paragraph so as to remove any ambiguity/doubts surrounding the information being conveyed. Each point could be placed in its own paragraph. In section 2.2, the author made a bold statement on "talented people governing the village..." This point important as it might be seemingly is void of any credible citation. Additionally, it begs the question as to where exactly this might be occurring. Would need revisiting by the author. In the final paragraph of Section 2, the author in attempting to make a case in respect of "The nature of duties and powers... seemingly did not present a clear harmonization of thoughts. This made this point appear disjointed and out of place. This too warrants rephrasing in alignment with relevancy of the section it falls under. Section 2.3, though a god showcase of the theoretical premise upon which the paper is written, again the author has been guilty of not citing correctly/not citing at all. | 1. We have added a sentence in the introduction (lines 51-59) to clarify the role of village heads, including their function in disseminating knowledge to the villagers. 2. Thank you for your valuable suggestion; we have supported our assertions and suppositions with relevant literature. 3. The introduction now elaborates on the role of village heads, particularly in lines 50-59. Additionally, we emphasize the importance of studying rural areas and the specific selection of our research subjects in the third paragraph. Your concerns are also addressed in the Methods section under "Study Subjects" (lines 118-130). 4. We have conducted a comprehensive revision of the introduction to ensure structural consistency. Following Reviewer 2's recommendation, we merged the introduction with the literature review, defining the significance of the research while providing a concise overview of key literature. 5. We have cited data from UNESCO in the introduction to underscore the severity of educational issues faced by students (line 43). Controversial assertions are now supported by appropriate references. 6. For any contentious assertions, we have provided supporting literature. 7. Thank you for your suggestion; we have rewritten this section accordingly. In line with Reviewer 2's advice, we merged the literature review with the relevant citations, actively addressing the corresponding issues during this process. 8. We have made significant adjustments to this section, removing any inappropriate statements. |
| **Methods**   1. To this end the author is best advised to consider having a section in the manuscript devoted to the study population and giving us a clear background of what it is about the population that is special and warrants it being investigated by way of this study. 2. Under section 3 (Research Design), it would augur well for good reading should the author include an area to address the study population specifically. 3. Under section 3.2.2, it is felt that the first two sentences though a bold statement appear not to be cited. In the absence of citations here, the question asked is whether the author is presenting an opinion or a mere conjecture. 4. Within the first paragraph of section 3.2.2, the author suddenly speaks of an econometric model and standardization, words of which connotes methods of analysis. Perhaps the author may opt to include a section that addresses specifically what these forms of analyses entail. Having a section specifically devoted to the methodology/methods employed could address this. This section could also include the kinds and types of analyses needed instead of morphing analysis with results. 5. Under section 3.2.3, again the author though referring to ''drawing on literature" fails to at the least cite one literature that that adopts the approach of controls for gender in the manner expressed in the manuscript. This is a n area for improvement by the author. the author is advised to at the least provide a basis/source for certain statements made or being alluded to. 6. Under section 4 the author appears to make reference to several kinds of analyses underpinning the results. However, what is not entirely clear is what are some of the key assumptions upon which these analyses are based. This need to be borne out in this section. 7. Additionally, re: paragraph that speaks of stepwise regression, the author in closing attempts to morph results with discussion/implications of work. The author is best advised to include this in the discussion/conclusion section of the manuscript. | 1. We have added a subsection in the Methods section specifically dedicated to describing the study population (Study Subjects, lines 118-130). 2. The "Study Subjects" subsection in the second part of the Methods provides a detailed introduction to the research population. 3. We have removed the inappropriate statement. 4. Thank you for your suggestion! This section includes variable definitions, and we acknowledge that we employed a straightforward approach. We have now consolidated this information in the Methods section. 5. We have provided relevant literature to support the rationale for the selection of control variables. 6. In the "Baseline Estimation Model" subsection, we have explained the assumptions of the model. 7. Additionally, we have moved the discussion of the stepwise regression results to the Discussion section (lines 238-246). |
| **Discussion**   1. In section 4 (e.g section 4.4.1) of the manuscript, the author is often guilty of beginning to speak of a method of measurement/analysis but then in the same breath talks of a discussion. This disjointedness ought to be addressed. An explanation of a methodology cannot suddenly descend to a discussion of something else that appears unrelated. | 1. We have revised this subsection as requested. The methods-related content has been placed in Section 2, specifically in the "Data Analysis Methods" subsection, while the discussion-related content is now included in Section 4, the "Discussion." |
| **Conclusion** |  |
| **References** |  |
| **Additional Comments**   1. The manuscript was void of page numbers and line numbering（）. This made the process of tagging areas of concern rather difficult for the reviewer. 2. The article title could be more specific/definitive and self-explanatory without having to refer to the body of the manuscript should the author choose to identify the study population therein. 3. The author is best advised to choose a single citation style instead of using two distinct citation styles at the same time. This flaw is very prominent in the introduction section of the manuscript. In other areas of the manuscript the author is guilty of alternating between citation styles. The author should seek to be consistent by using a single citation style. | 1. Thank you for your reminder. We have added page numbers and line numbers to the manuscript, and we apologize for any inconvenience this may have caused during your review. 2. We have revised the paper’s title to make it more self-explanatory: "The Impact of Village Heads' Educational Levels on Adolescent Academic Performance: Evidence from Rural China." 3. In accordance with the journal's requirements, we have standardized the reference style to "Vancouver." |

**Reviewer 2：**

| **Comments to the Author** | **Response and Revison** |
| --- | --- |
| General comment: I commend the authors for their innovative research ideas. However, the manuscript is quite lengthy and would benefit from a more conventional structure.  **1.** (e.g., abstract, introduction, methods, results, discussion, and conclusion). Adopting this format and streamlining the content could enhance overall clarity and coherence, reducing potential confusion for readers. | Dear Reviewer 2:  Many thanks for you insights and constructive comments. We hope the revisions we made have improved the paper and were able to address your concerns. The changes are highlighted in **red** in the manuscript file. Thank you !   1. Following your suggestion, we have restructured the manuscript to include the following framework: abstract, introduction, methods, results, discussion, and conclusion. |
| **Abstract**   1. I recommend consistently using the term 'China Family Panel Studies' throughout the abstract and methods sections rather than using 'China Family Tracking Survey’ in the abstract. | 1. Thank you for pointing out the issue. We have made revisions to the abstract. |
| **Introduction**  **1.**The literature review section is overly lengthy, and since this is not a systematic review, a concise summary would be more appropriate. I recommend merging the literature review with the introduction to create a focused and coherent introduction (specifically, 3 to 4 pages or less) that effectively summarizes the background, research context, and study hypothesis. | 1. We have combined the citations and literature review into a single section, and we hope the new version meets the standards of academic writing. |
| **Methods**   1. Table 1: Categorical variables, including gender, political identity, agricultural production, and internet use, should not be described using mean and standard deviation (SD), as these summary measures are appropriate only for continuous data with a normal distribution. Instead, categorical data should be presented using frequencies and percentages. | 1. We have corrected the previously erroneous expression (line 226). |
| **Results**   1. Tables: I recommend naming the columns as Model 1, Model 2, and Model 3 etc., instead of (1), (2), and (3). 2. Tables: I understand that the significance levels of 1%, 5%, and 10% correspond to p-values of 0.01, 0.05, and 0.1, respectively. However, most research considers associations with p-values below 0.05 as statistically significant. Therefore, treating p-values of 0.1 as indicating a significant association may be unconventional and should be carefully justified. 3. To better understand the data, it is more appropriate to present the coefficients and their 95% confidence intervals. 4. All tables should be self-explanatory. For example, in Table 5, Column 1: Edu_year 0.111***(0.023) – it's unclear what the numbers in brackets represent. Please clarify this in the table or the table notes. | 1. We have standardized the terminology to use "Model 1" and "Model 2." 2. We have revised the expression of significance for all results. The manuscript consistently indicates that only results with p < 0.05 are considered significant. Additionally, we have provided explanations for the symbols * and ** in the footnotes of the tables. 3. All results now include 95% confidence intervals, reported in brackets, for example, [0.0356, 0.226]. 4. Explanations are provided at the bottom of all tables. |
| **Discussion** |  |
| **Conclusion**   1. The conclusion is too lengthy. Including detailed discussions of mechanisms or extending the discussion detracts from the main points. This section should be concise, focusing exclusively on the study's major findings. 2. Policy recommendations: This section would be more effective if it informed the reader about the current policy and clearly explained how the study's findings could change, influence, or improve the existing policies. The current version lacks focus | 1. We have revised the conclusion to ensure it is concise and focused on the main findings. 2. We have emphasized the role of the "College Student Village Official" policy in China and how it can contribute to improvements in this area. |
| **References** |  |
| **Additional Comments**   1. The manuscript contains a mixture of citation styles. I recommend using a single citation style consistently throughout the manuscript—either Vancouver or Harvard—according to the journal's guidelines. 2. The term 'highly educated' usually refers to individuals with advanced degrees, such as a PhD or master's, who have specialized knowledge and skills. Since village leaders in China are unlikely to hold such qualifications, I recommend using the term 'well-educated' throughout the manuscript instead. If the authors have a compelling justification for using 'highly educated,' they may retain it, but this should be clearly explained. 3. I recommend restructuring the article to follow this sequence: abstract, introduction, methods, results, discussion, and conclusion. The current format presents the discussion before the results (i.e., in the literature review section), which can be confusing for readers. Additionally, combining the discussion with the results further adds to the confusion. A more precise separation of these sections would improve readability and coherence. 4. Adding line numbers to the manuscript would simplify the review process, allowing for more precise referencing and feedback. | 1. In accordance with the journal's requirements, we have standardized the reference style to "Vancouver." 2. Thank you for pointing out this issue; we have changed "highly educated" to "well-educated" throughout the manuscript. 3. Following your suggestion, we have reorganized the manuscript in the following order: abstract, introduction, methods, results, discussion, and conclusion. 4. We have added page numbers and line numbers, and we apologize for any inconvenience this may have caused during your review. |
