# Supplementary material for: The impact of village heads’ educational levels on adolescent academic performance: Evidence from rural China
Source: PLoS One. 2025 Jan 2;20(1):e0315822. doi: 10.1371/journal.pone.0315822 (PMC11694985; doi:10.1371/journal.pone.0315822)
Supplement: Attachment — Submitted filename: Response to reviewers.docx [file pone.0315822.s003.docx]

**Dear Editor and Reviewers,**

Thank you for the valuable comments and suggestions from both you and the reviewers, which have greatly enhanced our manuscript. Below, we provide a summary of the major revisions:

1. **References Check**: We conducted a thorough review of the references and confirmed that none have been retracted.
2. **Clarity and Wording Adjustments**: We made several changes throughout the manuscript to improve clarity, such as replacing terms to better align with the intended meaning (e.g., changing "control variable" to "covariate").
3. **Introduction and Discussion Revisions**: The introduction has been refined for improved flow, and the discussion has been streamlined. Each key research conclusion is now discussed in a separate paragraph, and the discussion is structured in four parts: findings, discussions of findings in relation to existing literature, strengths and limitations of the study, and implications of findings.
4. **Repositioning and Deletion**: We repositioned sections such as the ethics statement for better flow, and removed inappropriate or redundant statements where necessary.
5. **Robustness Checks and Conclusion Refinement**: We expanded on the robustness checks in the methodology section and condensed the conclusion, emphasizing the key findings and future research directions.
6. **Formatting Correction**: We apologize for the formatting oversight in Model 3, which caused discrepancies in reported significance levels. We have corrected this error (Line 281) to ensure consistency.

Please refer to the detailed responses below and the revised manuscript for specific modifications based on the reviewers' comments. We appreciate your thorough consideration and are grateful for the opportunity to improve our work.

We hope this revised manuscript has addressed your concerns, and look forward to hearing from you.

Thanks again!

***Editor**

**1.**Please review your reference list to ensure that it is complete and correct..

Response: We have conducted a thorough review of the references and have confirmed that none of the cited articles have been retracted.

***Reviewer 1**

**Thank you very much for your revision suggestions. These comments have significantly improved the clarity, accuracy, and precision of the manuscript. I will now address each of your suggestions in detail.**

1. Line 26- Author should relook at words 'well education levels. This does not read well. I take it that this might just be a typographical error.

Response: In Line 26, we have revised the wording to "well-educated."

.

2. Line 50- Authors may give consideration to linking rural areas of developing countries with the previous paragraph for the sake of coherence.

Response: Thank you for your suggestion. We have added a sentence at the beginning of the paragraph: "In light of these disparities, the role of village heads in rural areas of developing countries becomes increasingly significant" (Line 49). This addition helps to connect rural areas in developing countries to the previous paragraph.

3. Line 58- Consider inserting a full stop after the word capabilities. Next sentence should start with 'Those".

Response: In Line 57, we made modifications to improve the clarity of the language.

4. Lines 111-115 -suggest that the study employed a quantitative research design, however from the last few sentences of the introduction one gets the impression that there were both qualitative and quantitative approaches involved. Authors need to clarify.

Response: The previous version indeed had the potential to mislead readers. We have made the following modifications to the methodology section (Lines 85-87): "We used a cross-sectional study design to examine the association between village heads' educational levels and adolescents' academic performance, utilizing data from the CFPS to assess how these educational levels influence academic achievements." Additionally, we revised the introduction, specifically by removing the last few sentences.

5. Line 76-106- Authors appear to be guilty of discussing implications of the study findings at this stage of the article. Authors may want to shift these elements to the discussion section of the paper and perhaps close the introduction section with just the rationale for the study.

Response: Thank you for your suggestion. We have revised the introduction for improved flow and clarity, and, following your advice, concluded it by emphasizing the fundamental principles of our study (Lines 68-82). The content that was previously more suited for discussion has now been relocated to the discussion section.

6. Line 107-109 Not sure if this is warranted as the section head will bring this out..

Response: We have removed the inappropriate statement.

7. Line 112- this paragraph could be restructured differently to eliminate the obvious repetitiveness in the presence of the second sentence. Not sure of the reason the author identifies quantitative assessment again when this was already stated in the first sentence.

Response: We have made significant adjustments to this section, removing any inappropriate statements (Lines 85-87). Specifically, we revised the text to: "We used a cross-sectional study design to examine the association between village heads' educational levels and adolescents' academic performance, utilizing data from the CFPS to assess how these educational levels influence academic achievements."

8. Line 134 -authors suddenly speaks of 'survey questionnaire'. Are the authors saying that the official channels of CFPS uses the survey questionnaire as a tool. If this is correct then the authors should state this by using words such as " The survey questionnaire a tool used in the ....... includes....This would make for better reading and lend to coherence..

Response: In Lines 105-108, we made the following modification: "The survey questionnaire, used as part of the CFPS, includes multiple-choice questions and scales that cover information on family background, educational environment, and characteristics of village heads."

9. Line 144- authors need to qualify the term basic characteristics. Are they referring to demographics, socio-demographics, clinical...what exactly?

Response: In Lines 113-115, we made the following modification: "The 2014 CFPS dataset includes basic characteristics, such as the economic and social conditions of 365 villages, as well as demographic information about the village heads, along with data on the academic performance of adolescents within these villages." This revision provides a clearer definition of the term "basic characteristics."

10. Line 148- Authors may wish to reposition the ethical statement at the end of the Methods section instead of having t here.

Response: We have repositioned the ethics statement to the end of the methodology section (Lines 270-276).

11. Line 168- what was the approach used in standardizing test scores.

Response:My apologies for the oversight. We have standardized the test scores using z-scores based on the children's current educational level. In Lines 130-132, we provided the following explanation: "However, more educated children might achieve higher scores, so this paper standardizes the test scores based on the children's current educational level using z-scores to reduce intergroup differences."

12. Line 197- Control variables. Are the authors saying that they controlled for confounding and that these were potential confounders. What exactly is the rationale for control variables.

Response: We apologize for any confusion caused. The term "control variable" is used in economics and corresponds to "covariate." We have revised it to "covariates." The selection of covariates was based on a priori theoretical considerations. The related modifications can be found in Lines 160-185.

13. Line 229- Can authors say what excatly is meant by conduct robustness checks. What was done exactly? Lines 327-330: If this is relatable to what was stated in line 229, then authors would need to find a way to bring section together under methods. Would suggest that we not repeat methodology under results section. If we are reporting on results, then report on results and not repeat method.

Response: In our robustness checks, we aimed to verify the stability and reliability of our results by using alternative model specifications and different sample subsets. We have moved the content originally in Lines 327-330 to the methodology section. This part explains why we conducted the robustness checks and specifically what methods were used (Lines 229-244).

14. Line 364-366: similar occurrence as in lines 327-330, where the authors in presenting the results of mechanism analysis appear to to be also describing the methods involve. Authors should review and ensure that only results are captured.

Response: We have removed this inappropriate statement.

15. Line 394: why are the authors discussing or referencing discussions above when there is a section wholly devoted to discussions (line 415). Authors should review and make a decision on what should be in results and what should be under discussions..

Response: We apologize for the confusion. Our initial intention was to inform readers about the purpose of this section, but the wording was problematic. We have now removed this part in the current version.

16. Line 416, 437, 464: Authors may wish to refine these subheadings by taking out the word 'discussion' since the heading at line 415 already covers this. Further authors are asked to consider presenting the results in the format of

-findings (discuss all findings)

-discussions of findings in relation to existing literature

-strengths and limitations of the study

-implications of findings

This approach will significantly strengthen the paper and make for easy reading..

Response: Thank you for your suggestion. We have now streamlined the discussion section, ensuring that each research conclusion is discussed in a separate paragraph. Additionally, we structured our discussion according to the following components: findings, discussion of findings in relation to existing literature, strengths and limitations of the study, and implications of the findings. The related modifications can be found in Lines 380-450.

17. Line 494: Very good points, however, this section reads more like what the discussion section should read like or consist. The authors could tighten up the conclusion to produce at the most two significant paragraphs that speaks to key but impactful points emanating from the findings but are also overarching.

Response: In the current version, we have further condensed the conclusion section. We summarized the key findings and their significance while emphasizing directions for future research (Lines 452-464).

***Reviewer 2**

**Thank you for pointing out this issue. We have carefully reviewed all statistical analyses to ensure that similar issues do not occur, and we have also made revisions to the conclusion section. I will now address each of your suggestions in detail.**

1. In my previous review, I suggested adding 95% confidence intervals. In Model 2, the results now show: -0.106* [-0.230, 0.018]. The 95% confidence intervals indicate that the range includes zero, which means the result is not statistically significant at the 5% level. Please check if this is a typographical error and revise accordingly.

Response: We sincerely apologize for the formatting oversight. This was an error, and it should be consistent with the significance level reported in Model 3. We have corrected this issue (Line 281) based on your comment regarding this problem.

2. In my previous feedback, I advised against extending the discussion into the conclusion section. It seems that this point was not fully addressed. For example, in line 511, there is a citation of other studies, which is not appropriate for this section. The Conclusion should not discuss findings or introduce new informationt.

Response: We have rewritten the conclusion, focusing only on the main findings of the study and directions for future research (Lines 452-464).
