# Supplementary material for: The impact of village heads’ educational levels on adolescent academic performance: Evidence from rural China
Source: PLoS One. 2025 Jan 2;20(1):e0315822. doi: 10.1371/journal.pone.0315822 (PMC11694985; doi:10.1371/journal.pone.0315822)
Supplement: Attachment — Submitted filename: Response to reviewers.docx [file pone.0315822.s004.docx]

**Dear Editor ,**

Thank you for your positive feedback regarding our revisions, and for the opportunity to further improve the manuscript. We are grateful for the valuable comments provided by both you and the referees, and we appreciate the detailed consideration given to our work.

Regarding your additional comment about the justification for the choice of instruments, we have made the following clarifications and revisions:

**First**, concerning the choice of research instruments, we have added references to substantiate our use of the China Family Panel Studies (CFPS) survey data. Specifically, we have cited relevant literature demonstrating that the CFPS is widely used and accepted in academic research, particularly in economic studies. For instance, research utilizing CFPS data has been published in leading economics journals such as the American Economic Review, and articles in PLOS ONE have also utilized CFPS data to support their findings. These references are now included in **lines 109 and 110** to emphasize the robustness and validity of using CFPS data in our study.

**Second,** we understand that there may have been concerns regarding the instrumental variables employed in our analysis. To address this, we have expanded on the rationale for the selection of our instrumental variables. In econometric and social science research, instrumental variable (IV) methods are commonly used to mitigate endogeneity issues, thereby enhancing the accuracy of the study's conclusions. Specifically, we used community-level averages as instruments for individual-level variables, which is a standard approach in the field. We have supported this choice by including citations from authoritative sources, including the work of the renowned econometrician Jeffrey Wooldridge, to further substantiate our methodological choices. These additions can be found in **lines 218-230** of the revised manuscript.

In addition, we have addressed the two errors that reviewer pointed out. Specifically, we have corrected the word separation in line 85 from "across-sectional" to "a cross-sectional". We have also inserted the appropriate citation at the end of the sentence on "Karadag's..." in lines 396.

We hope that these additions and clarifications address your concerns satisfactorily. Additionally, we acknowledge that we may have misunderstood your reference to the 'choice of instrument', but we are confident that all the research instruments used in the manuscript are reliable. We are happy to provide any further revisions or additional details if needed. Once again, we greatly appreciate your constructive feedback and the opportunity to finalize this manuscript for publication.

**Thank you for your consideration.**

**Best regards,**

Jing Li

Huan Deng

Jun Li
